# Capsaicin-Sensitive Vagal Afferent Nerve-Mediated Interoceptive Signals in the Esophagus

**DOI:** 10.3390/molecules26133929

**Published:** 2021-06-28

**Authors:** Mingwei Yu, Crystal Chang, Bradley J. Undem, Shaoyong Yu

**Affiliations:** Department of Medicine, Johns Hopkins University School of Medicine, Ross Research Building, 720 Rutland Ave, Baltimore, MD 21205, USA; mingweiyu11@gmail.com (M.Y.); cchan121@jhu.edu (C.C.); bundem@jhmi.edu (B.J.U.)

**Keywords:** capsaicin, esophagus, vagal afferent, nodose, jugular, nociception, interoception

## Abstract

Heartburn and non-cardiac chest pain are the predominant symptoms in many esophageal disorders, such as gastroesophageal reflux disease (GERD), non-erosive reflux disease (NERD), functional heartburn and chest pain, and eosinophilic esophagitis (EoE). At present, neuronal mechanisms underlying the process of interoceptive signals in the esophagus are still less clear. Noxious stimuli can activate a subpopulation of primary afferent neurons at their nerve terminals in the esophagus. The evoked action potentials are transmitted through both the spinal and vagal pathways to their central terminals, which synapse with the neurons in the central nervous system to induce esophageal nociception. Over the last few decades, progress has been made in our understanding on the peripheral and central neuronal mechanisms of esophageal nociception. In this review, we focus on the roles of capsaicin-sensitive vagal primary afferent nodose and jugular C-fiber neurons in processing nociceptive signals in the esophagus. We briefly compare their distinctive phenotypic features and functional responses to mechanical and chemical stimulations in the esophagus. Then, we summarize activation and/or sensitization effects of acid, inflammatory cells (eosinophils and mast cells), and mediators (ATP, 5-HT, bradykinin, adenosine, S1P) on these two nociceptive C-fiber subtypes. Lastly, we discuss the potential roles of capsaicin-sensitive esophageal afferent nerves in processing esophageal sensation and nociception. A better knowledge of the mechanism of nociceptive signal processes in primary afferent nerves in the esophagus will help to develop novel treatment approaches to relieve esophageal nociceptive symptoms, especially those that are refractory to proton pump inhibitors.

## 1. Esophageal Interoception and Nociception

Interoception is defined as “the representation of the internal world and includes the processes by which an organism senses, interprets, and regulates signals from within itself” [1]. The concept of “interoceptive” was first described by Dr. Sherrington over a hundred years ago. He referred to interoceptive as the internal surface of the body in contrast to exteroceptive as the external surface in direct contact with the environment [2]. A representation of one’s internal world is often initiated by activation of afferent nerves that are sensitive to certain physiological activities in visceral organs that are derived largely from sensory neurons situated mainly in the dorsal root ganglia and vagal sensory ganglia [1]. Beyond these types of general proclamations, relatively little is known about the afferent nerve subtypes, especially vagal afferents, in mediating interoceptive signals.

The term nociceptor, or *noci-receptor*, was coined by Sherrington as the neural apparatus responsible for detecting a noxious stimulus [2,3]. Nociceptors are now known to be a subpopulation of unmyelinated slow conducting polymodal C-fibers that are found to innervate all somatic and visceral tissues. A noxious stimulus was considered to be any harmful exteroceptive stimulus that may cause tissue injury [4]. Activation of afferent nociceptors initiates the process by which intense thermal, mechanical, or chemical stimuli are detected. The ensuing action potential discharges are conducted to the central nervous system, where the information is processed into sensations and reflexes aimed at reducing tissue damage [5]. A common consequence of stimulation of nociceptors in the somatosensory system is acute pain. This has led to nociceptors being defined as pain nerves. This definition, however, is too confining, as many sensory nerves that are activated by noxious stimuli do not lead to acute pain. Activation of visceral nociceptors may lead to sensations of discomfort but can also induce strong defensive reflexes, such as coughing and sneezing, as well as strong autonomic reflexes aimed at ridding the host of the noxious agent in the absence of acute pain [6].

In contrast to somatosensation that is exclusively processed by spinal afferent nerves, the internal signals from visceral tissues and organs are processed by both spinal afferents and vagal afferents [7,8,9]. It is now recognized that vagal afferent nerves are interoceptive nerves that can encode visceral stimulus not only in physiological but also in noxious ranges [6,10]. A better understanding of interoceptive signaling through primary visceral afferents will improve our knowledge of the pathogenesis of many gastrointestinal sensory/motor dysfunctions.

Stimulation of esophageal afferent nerves can lead to the sensation of the presence of food or fluid that helps to coordinate swallowing. Activation of nociceptors in the esophagus usually presents as heartburn and non-cardiac chest pain, the two most common symptoms in many esophageal disorders. Heartburn and non-cardiac chest pain are unlike typical somatic pain sensations, which are processed exclusively through primary afferent neurons in the dorsal root ganglion (DRG). Classic nociceptive C-fibers in the esophagus are derived from neurons situated in three disparate ganglia: the DRG, the nodose vagal ganglia, and the jugular vagal ganglia. It is likely that all three subtypes of esophageal nociceptors are involved in interoception and in coordinating sensations (pain) and reflexes (secretions) aimed at avoiding and reducing potential tissue injury. A more granular understanding of the specific roles played by each of these three nociceptor subtypes is lacking. In this review, we summarize the recent progress on vagal afferent neurobiology in the esophagus with specific emphasis on the subtypes of afferents that are able to process interoceptive signals, which may subsequently lead to nociception. 

## 2. Esophageal Primary Afferent Pathways

Esophageal sensory transduction is initiated by the stimulation of primary afferent neurons at their peripheral nerve terminals, which are distributed in the wall of the esophagus. The evoked action potentials (APs) are transmitted through both spinal and vagal afferent pathways to their central terminals, which synapse with the neurons in the central nervous system [7,8,9]. Activation of these afferent nerves with non-noxious stimuli can lead to normal sensations of the presence of food or fluid that likely subconsciously trigger peripheral and central reflexes to coordinate motor functions. In contrast, noxious stimulation and stimuli produced at sites of local inflammation can activate nociceptors that could generate pain and nocifensive behavior in order to avoid potential tissue damage. 

**Figure 1:**
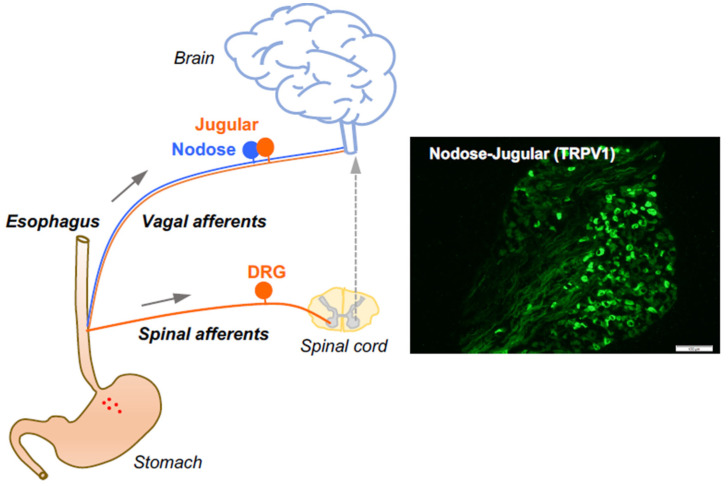
Esophageal primary afferent pathways. The extrinsic primary afferent nerves in the esophagus include vagal and spinal afferents with their neuronal cell bodies in nodose and jugular ganglia (NJG) and dorsal root ganglia (DRG), respectively. (right: TRPV1 immunoreactivities in mouse NJG, bar size = 100 μM).

The afferent fibers in the esophagus comprise low threshold mechanosensitive nerves that exquisitively respond to a mechanical tension. These so-called “tension receptors” are strongly activated by mechanical distention at pressures that are not sensed as pain. They are derived from neurons in the nodose ganglia and are characterized by conduction of action potentials in the Aδ range (about five times faster than that of nociceptive C-fibers), indicative of small, myelinated nerves. The rest of the afferent innervation of the esophagus comprises polymodal unmyelinated C-fibers that have all the characteristics of classically defined nociceptors. As previously mentioned, the esophagus is innervated by at least three distinct subtypes of nociceptive afferent nerves. The characteristics of the nociceptive subtypes depend strictly on the location of their neuronal cell bodies. The nociceptor subtypes include vagal C-fibers derived from cell bodies in the vagal nodose ganglion (vagal placodal C-fibers) and jugular ganglion (vagal neural crest C-fibers) [10], in addition to C-fibers with cell bodies in the spinal DRG (spinal neural crest C-fibers) [11]. The jugular and DRG C-fibers innervating the esophagus are both neural crest-derived and share many phenotypic features. Therefore, they are both substantially distinct from the placodal-derived nodose C-fibers. Based on their activation profiles, all three of these nerve subtypes fit Sherrington’s definition of a nociceptor, thereby serving the function to, in Sherrington’s words, “provide the (organ) with a so-to-say sense of its own potential injury” [2]. The vast majority of nociceptive C-fibers innervating the esophagus express TRPV1 and can be activated by capsaicin [10,11] (Figure 1). Here, we focus on the roles of capsaicin-sensitive vagal afferent C-fibers in processing esophageal interoceptive signals and their potential contribution to esophageal nociception (heartburn and non-cardiac chest pain).

## 3. Capsaicin-Sensitive Vagal Afferent C-Fibers in the Esophagus

It is critical to define the functional difference between vagal nodose (placode-derived) and jugular (neural crest-derived) neuron populations. This is because these two neuron populations innervate different parts of the CNS. Nodose fibers synapse with neurons in the nucleus tractus of solitarius (NTS) of the brain stem, whereas it has recently been shown that jugular fibers synapse with neurons in the paratrigeminal nucleus, similar to somatosensory nociceptors [12]. Thus, the subsequently induced sensations and reflexed autonomic efferent activities will depend on whether nodose or jugular C-fibers are activated.

Nociceptive C-fiber neurons can be further divided into subpopulations by their expression of distinctive neuropeptides, receptors, and ion channels that encode specific noxious thermal, mechanical, and chemical stimuli [4]. For example, tachykinergic C-fibers that innervate the esophagus, which are generally derived from neurons in the DRG and vagal jugular ganglion. Most nodose C-fibers are non-tachykinergic. Therefore, one can argue that any substance P-mediated inflammation, so-called neurogenic inflammation, in the esophagus, is more likely caused by activation of DRG and vagal jugular C-fibers rather than nodose C-fibers.

The location of afferent nerve endings in different layers of the esophageal wall may reflect their specific functional roles. The afferent nerve Aδ-fibers are usually distributed in the muscle layers and form intraganglionic laminar endings (IGLEs) and intramuscular arrays (IMAs) with the myenteric plexus to serve as tension and stretch receptors. In contrast, nociceptive C-fiber free nerve endings often terminate in the mucosal and submucosal layers to sense potential tissue damage by responding to noxious thermal, mechanical, and chemical stimuli in the lumen [13,14]. At present, the distribution of nodose vs. jugular C-fiber nerve endings in the esophagus is still less clear. Using in vivo transduction of vagal nodose and jugular ganglion neurons respectively by adeno-associated virus (AAV2-eGFP) vectors, a recent study demonstrated that neural crest-derived jugular afferent nerve terminals are richly distributed in esophageal mucosa in contrast to placode-derived nodose afferent nerve endings that more deeply innervated in the submucosal layer [15]. Therefore, noxious chemicals in the lumen are more likely to activate the neural crest C-fibers (DRG and vagal jugular nociceptors) than the placodal C-fibers (nodose nociceptors). However, some of this discrimination may be lost when the epithelial barrier function of the esophageal mucosa is damaged.

## 4. Activation and Sensitization of Esophageal Vagal Nodose and Jugular C-Fibers

**Figure 2:**
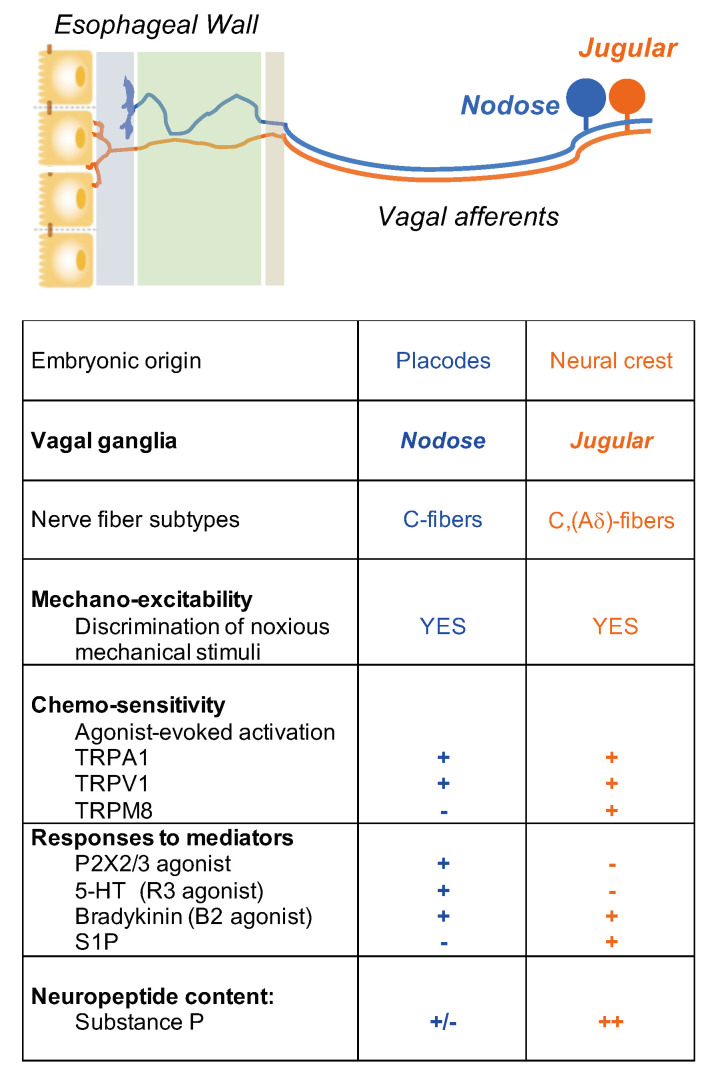
Nociceptive vagal afferent subtypes in the esophagus.

Electrophysiological studies have revealed functional distinctions between the two vagal C-fiber subtypes in the esophagus. We have successfully established an ex vivo esophageal-vagal preparation for extracellular single-unit recordings [10]. In this model, the recording electrode is placed into intact vagal nodose or jugular ganglion, respectively, and the evoked action potential discharges are recorded in a sensory neuron cell soma, while noxious chemical and mechanical stimuli are applied at its nerve terminals in the esophagus. This unique approach enables us to thoroughly characterize esophageal vagal afferent nerve subtypes, to define the key ion channel-mediated activation responses to noxious stimuli, and to reveal the mechanisms of activation and sensitization [16,17,18,19,20,21] (Figure 2).

Many stimuli in the esophagus can activate both nodose and jugular C-fibers, including tissue distension in physiological and noxious ranges and all substances that can gate either TRPV1 [10] and TRPA1 [18]. Chemical stimuli with certain inflammatory mediators have been identified to selectively activate nodose but not jugular C-fibers. For example, vagal nodose C-fiber neurons express both P2X3 and P2X2 receptors and are strongly activated by ATP, whereas neural crest jugular and DRG C-fibers only express P2X3 receptors and are categorically not activated by ATP [10,11]. This argues for the hypothesis that stimulation of the heteromeric P2X2/3 receptor provides enough current to depolarize the terminal membrane to the voltage threshold of voltage-gated sodium channels. Current through the homomeric P2X3 receptors, on the other hand, is insufficient to evoke action potential discharge. The same situation exists with respect to vagal C-fibers in the respiratory tract [22,23,24]. Like ATP, serotonin via the 5-HT3 receptor [16] and adenosine via the A2a receptor [25,26] selectively activated nodose but not jugular C-fibers. In contrast, TRPM8 agonist WS-12 and inflammatory mediator sphingosine-1-phosphate (S1P) selectively activate esophageal jugular but not nodose C-fibers [27,28]. Bradykinin activates and sensitizes both nodose and jugular C-fibers in the esophagus [18]. Our recent study [23] characterized transcript expression profiles of inflammatory mediator receptors in TRPV1-positive vagal afferent C-fiber neurons in the mouse airway. Similar profiles in the esophagus deserve further investigation, as it could be helpful to apply genetic tools to better clarify the interactions of TRPV1 and mediators. 

Visceral sensory afferent nerves sense stimuli in healthy and inflamed tissues differently. In inflammation and tissue injury conditions, their sensitivity is enhanced by a large variety of inflammatory mediators in the extracellular space, leading to peripheral sensitization. Sensitization of the peripheral terminal occurs when a stimulus interacts with its receptor in a manner that may not activate the nerve fiber but causes the excitability of the terminal to increase such that a stimulus that was previously subliminal now leads to action potential discharge. A sensitized terminal can also lead to cases in which the frequency and duration of the action potential discharge are increased. The process of peripheral sensitization often involves certain inflammatory mediators interacting with their selective receptors in the sensory nerve terminal membrane leading to changes in the excitabilities of key ion channels [4,5]. Peripheral sensitization can lead to increases in the activity of the central nerve terminals and cause a central sensitization in which both quantitative and qualitative changes occur in the central processing. Central sensitization has been well studied in the somatosensory system, where it is thought to play an important role in hyperalgesia and allodynia [4,5]. Although much less investigated, clinical studies indicate that acid reflux may induce central sensitization in patients with unexplained chest pain [29]. Nociceptor activation along with peripheral and central sensitization can cause the nocifensive nervous system to function pathologically and initiate host-defense, which may lead to many of the key symptoms of acid reflux and inflammatory esophageal disorders. Here, we summarize the activation and/or sensitization effects of two key inflammatory cells, namely eosinophils and mast cells, as well as the effects of certain mediators on TRPV1-expressing vagal afferent C-fiber subtypes in the esophagus. 

## 5. Potential Roles of Capsaicin-Sensitive Afferents in Esophageal Disorders

### 5.1. Eosinophils

Eosinophils, by definition, are elevated in esophageal mucosa in the eosinophilic esophagitis (EoE), as well as other inflammatory esophageal disorders. Eosinophils can release a variety of cationic proteins that may influence afferent C-fiber function in the proximity. It has been shown that eosinophil cationic protein and major basic protein sensitized airway vagal C-fibers. Such effects could be prevented by pretreatment with poly-l-glutamic acid (PLGA) to neutralize cationic charges [30,31]. In esophageal nodose C-fibers, perfusion with synthetic cationic protein poly-l-lysine (PLL) did not evoke action potential discharges but increased their responses to esophageal distension. This potentiation effect could be prevented by pretreatment with PLGA. In contrast to nodose C-fibers, PLL neither induced action potential discharges nor changed the responses to esophageal distension in jugular C-fibers [32]. The roles of eosinophil granule proteins in the regulation of esophageal vagal afferent nerve mechano-excitabilities deserve further investigation. The sensitization of mechanical activity, in theory, could lead to a situation in which the action potential frequency that is usually interpreted as innocuous by the CNS is now increased to a point where the same innocuous distention is perceived as noxious, setting in motion the process by which the sensations and reflexes become troublesome. 

The effect of allergic esophageal inflammation on nodose C-fibers’ response to acid was further addressed in an allergen-sensitized and in vivo challenged guinea pig, eosinophilic esophagitis-like disease model. Acute ovalbumin inhalation in ovalbumin-sensitized guinea pig led to mast cell degranulation and eventually to an elevation in the numbers of infiltrated mast cells and eosinophils [33]. Chronic allergen-challenge in this model significantly increased acid responsiveness in esophageal nodose and jugular C-fiber neurons. Such increases were abolished by pretreatment with TRPV1 antagonist AMG-9810 [34]. Consistent with acid-elicited effects, allergic inflammation also led to increased expression and function of TRPA1, another important transient receptor potential (TRP) channel, in esophageal-specific nodose and jugular neurons [21]. Another interesting observation made in the guinea pig model of allergic EoE was a decrease in transepithelial resistance. This is relevant to the present discussion as it allowed an intra-esophageal infused TRPA1 agonist AITC to activate mucosal nodose and jugular C-fibers, whereas, in the healthy animals, AITC did not penetrate the barrier in sufficient quantities to activate these nerves [21].

### 5.2. Mast Cells

Mast cells are strategically distributed in close proximity to afferent nerve terminals in the host-defense surface and play a key role in sensitization of nociceptive C-fibers’ excitability [6,35]. Moreover, in esophageal allergic disorders, such as EoE, mast cell numbers are increased in the wall of the esophagus. The percentage increase in the number of mast cells can be similar to that of eosinophils. Our recent studies have systematically investigated how IgE-mediated mast cell activation sensitizes esophageal vagal afferent C-fibers, what roles mast cell mediators play, and which ion channels downstream mediate C-fibers’ hyper-excitability. In ovalbumin-sensitized guinea pig esophagus, ovalbumin perfusion selectively activated tissue mast cells to induce histamine release. This significantly enhanced esophageal vagal nodose C-fibers’ excitabilities to both esophageal distension and chemical stimulations. Such sensitization effect was long-lasting even after tissue histamine was washed out and could be prevented by histamine H1 receptor antagonist only before but not after mast cell degranulation [17]. The possible role of another preformed mast cell mediator, tryptase, has been investigated using the same approach. The study demonstrated that protease-activated receptor-2 (PAR-2)-activating peptide mimicked mast cell activation-induced long-lasting sensitization of esophageal nodose C-fibers. Such effects could be inhibited by pretreatment with TRPA1 inhibitor HC-030031, suggesting that TRPA1 plays a key role downstream to process PAR2-dependent sensitization of nodose C-fibers [19]. In addition to mast cell preformed mediators, histamine and tryptase, the role of the mast cell lipid mediator prostaglandin D2 (PGD2) in mast cell activation-induced sensitization was further clarified. PGD2 perfusion to nodose C-fiber nerve terminals in the esophagus did not evoke action potential discharges but significantly increased their excitabilities to esophageal distension. Such sensitization effect could be mimicked by prostaglandin D2 DP1 (PGD2 DP1) receptor agonist BW 245C. Pretreatment with PGD2 DP1 receptor antagonist BWA868C inhibited both mast cell activation- and PGD2 perfusion-induced sensitization effects on esophageal nodose C-fibers. Patch clamp recordings indicated that PGD2 could decrease the threshold of action potential discharges in esophageal-labeled nodose neurons. PGD2 perfusion did not activate or sensitize esophageal nodose Aδ-fibers [20]. 

### 5.3. TRPV1 and Acid

TRPV1 in the terminals of esophageal nociceptors can, in theory, participate in the activation of the nerves induced by capsaicin found in foods, endogenous capsaicinoids, acid, heat, and certain G-protein coupled receptors, such as bradykinin. Acid reflux-induced heartburn is the most common symptom in many esophageal disorders. At present, the primary afferent pathways that mediate this noxious stimulation are still less clear. The responses of capsaicin-sensitive vagal afferents to esophageal acid instillation have been investigated in healthy and inflamed esophagi by electrophysiological recordings in animal models. Using extracellular recording ex vivo in guinea pig esophageal-vagal preparations, acid perfusion in healthy esophagi did not evoke action potential discharges in nodose C-fibers but significantly increased their response to esophageal distension, which could be recovered after washing acid out for 90 min. In jugular C-fibers, acid perfusion not only evoked action potential discharges but also inhibited their response to esophageal distension thereafter, which was not recovered after washing out acid for 120 min. Pretreatment with TRPV1 antagonist AMG-9810 inhibited acid-induced effects in nodose and jugular C-fibers [36]. The different responses to acid between esophageal nodose and jugular C-fibers might result from the differences in their nerve terminal distributions in the esophagus. Esophageal nodose C-fiber nerve endings are mainly distributed in the submucosal layer, while jugular C-fiber nerve terminals are more superficially distributed in the mucosal epithelium [15]. This morphological feature makes jugular C-fibers more accessible to acid in the esophagus. 

In addition to the TRPV1 antagonist, the localized inhibitory effect of QX-314 on acid-evoked activation of esophageal capsaicin-sensitive C-fiber neurons was investigated. QX-314 is a positively charged derivative of lidocaine with a molecular mass of 263 Da. It is cell membrane impermeable and can block neuronal sodium channels only when applied intra-cellularly. A recent study demonstrated that activation of TRPV1 by capsaicin successfully delivered QX-314 through the open pore of TRPV1, which led to the blockade of sodium channels in TRPV1-positive DRG neurons [37]. Our recent study demonstrated that in the presence of acid, which is a TRPV1 opener, QX-314 inhibited acid-induced activation of esophageal jugular C-fiber neurons. This result supports a localized application of QX-314 in the esophagus to block esophageal nociception in acid reflux disorders [38]. 

The effect of acid on vagal afferent nerve function was also studied in a rat esophagitis model (fundus ligation). The number of capsaicin- and acid-responsive vagal afferents was significantly increased in esophagitis rats than controls. TRPV1 antagonist AMG-9810 could block capsaicin and acid-induced activation responses in these esophageal distension-sensitive vagal afferents [39].

In expression studies, TRPV1-positive nerves were identified within the lamina propria and the epithelium of the human esophagus by immunostaining. In patients with non-erosive reflux disease (NERD), total esophageal acid exposure time, but not heartburn symptom, was correlated with the density of immunoreactivity of TRPV1-positive fibers [40]. In patients with gastroesophageal reflux disease (GERD), the density of TRPV1-immunoreactive sensory nerve fibers was increased in the inflamed esophagus [41]. A recent study revealed that NERD patients had significantly increased expression of TRPV1 on superficial sensory nerves compared to those with erosive reflux disease and Barrett’s esophagus [42].

The Bernstein test (also called the acid perfusion test) can usually reproduce the symptom of heartburn. However, it may also lead to false-negative results due to the variable integrity of the epithelial barrier and diluted acidity that reaches the nociceptive nerve endings in the esophagus. The effect of capsaicin-evoked esophageal sensation and nociception has been evaluated in healthy human subjects. Intra-esophageal infusion with capsaicin-containing red pepper sauce suspension significantly decreased the thresholds of both perception and discomfort in response to esophageal balloon distension in healthy volunteers [43]. In healthy subjects who experienced heartburn after meals, capsaicin ingestion did not change the severity of standard meal-induced heartburn symptoms, but significantly decreased the time to reach the peak of heartburn score as measured at 15-min intervals within 7 h after a meal [44]. Intra-esophageal perfusion with an acid plus capsaicin solution significantly decreased the esophageal pain threshold to heat and electric stimuli and increased the referred pain area to mechanical and electric stimuli in healthy subjects [45]. To gain more access to the nerve endings, capsaicin injection into esophageal submucosal elicited severe heartburn and chest pain symptoms in healthy subjects, compared to acid injection that induced none or mild symptoms [46]. These observations indicate that capsaicin can directly activate sensory afferent nerve endings and sensitize/desensitize their responses to esophageal mechanical and chemical stimulations. Therefore, activation of TRPV1 in esophageal afferent C-fibers by acid and capsaicin and sensitization of TRPV1 by tissue mediators could be crucial to develop esophageal nociception and hyperalgesia. 

Functional magnetic resonance imaging (fMRI) has been applied to identify the specific cortical areas that responded to esophageal balloon distension and acid perfusion in human subjects [47,48,49,50]. Kern et al. reported that esophageal acid perfusion for 10 min in healthy subjects did not induce heartburn symptoms but significantly increased fMRI signal intensity. The activation latency, peak, and deactivation of such response differed from that evoked by esophageal distension [47]. This group further compared esophageal acid infusion-evoked cerebral cortical fMRI activities in GERD patients and healthy controls. Most GERD patients exhibited cortical activities along with heartburn. In contrast, healthy subjects developed cortical activities without any feeling of heartburn. The signal increase in GERD patients was greater than that of healthy controls, suggesting a possible central sensitization effect [29]. Validation of fMRI assessment on esophageal-specific cortical activities could be helpful to differentiate non-cardiac chest pain from angina. 

The effects of TRPV1 antagonist on heartburn and esophageal pain have been addressed by two clinical trials. In healthy subjects, TRPV1 antagonist AZD1386 significantly increased the pain threshold of esophageal heat stimulation but not that of other stimuli (including distension, electric current, and acid) [51]. However, in NERD patients, AZD1386 was shown to be less effective in changing the moderate pain thresholds to esophageal stimuli induced by heat, distension, and electric current [52]. Localized application and a more selective inhibitor could be alternative approaches to increase the efficacy and decrease the side effects of the TRPV1 antagonist. Here it should be kept in mind that TRPV1 is not the only transducer of acid-induced activation of acid. Certain acid-sensitive ion channels (ASICs) are also expressed by C-fibers and are likely to contribute to acid-induced activation of esophageal nociceptors [53].

### 5.4. Serotonin (5-Hydroxytrytamine, 5-HT)

5-Hydroxytrytamine (5-HT, serotonin) is one of the most enriched mediators in the gastrointestinal (GI) tract. Its effects on intrinsic enteric neurons and extrinsic vagal and spinal afferents in the GI tract have been extensively investigated and nicely summarized [54,55]. The effects of 5-HT on esophageal vagal nodose and jugular C-fiber neurons were compared by both extracellular recordings in ex vivo esophageal-vagal preparations and by patch clamp recordings in esophageal-labeled nodose and jugular neurons, respectively. 5-HT selectively activated esophageal nodose but not jugular C-fiber neurons. Such activation effect was mimicked by 5-HT3 receptor agonist 2-methyl-5-HT and could be prevented by 5-HT3 receptor antagonist ondansetron and Y-25130. In addition, 5-HT did not activate esophageal-labeled DRG neurons. The differing responsiveness to 5-HT helps to discriminate placode-derived vagal nodose C-fibers from neural crest-derived vagal jugular and spinal DRG afferent nerves in the esophagus [16].

### 5.5. Bradykinin

Bradykinin (BK) is cleaved from the plasma precursor kininogen by kininogenase during inflammation or tissue injury. BK, via its G protein-coupled receptor, not only activates afferent neurons but also sensitizes their response to other stimuli. The effects of BK on esophageal vagal afferent nerve subtypes have been compared and determined. BK activates most esophageal nodose and all jugular C-fibers. This activation is associated with a significant increase in response to esophageal distension. Such effects can be prevented by the BK B2 receptor antagonist WIN64338. TRPA1 agonist AITC activates most BK-positive nodose and jugular C-fibers. Pretreatment with TRPA1 inhibitor HC-030031 prevents BK-induced mechanical hyperexcitabilities but not BK-evoked activation responses in esophageal nodose and jugular C-fibers. In contrast, esophageal vagal nodose Aδ-fibers do not respond to BK or AITC and fail to show mechanical hypersensitivity after BK perfusion [18].

### 5.6. Adenosine

Clinical studies demonstrated that intravenous adenosine induced esophageal hyperalgesia by lowering the threshold of pain sensation induced by esophageal distension. Adenosine antagonist attenuated high-dose adenosine-evoked esophageal discomfort and chest pain [56,57]. These indicate that adenosine may regulate esophageal afferent nerve functions. The possible mechanism has been experimentally addressed. Single-cell RT-PCR analysis of mRNA expression in esophageal-labeled afferent neurons demonstrated that the majority of TRPV1-positive afferent neurons expressed adenosine receptors. The jugular and DRG C-fiber neurons mainly expressed adenosine A1 receptor, and nodose C-fiber neurons expressed adenosine A1 and A2A receptors. A functional study supported these expression profiles and demonstrated that adenosine evoked action potential discharges in both esophageal nodose and jugular C-fibers. Jugular C-fibers could be activated by A1 receptor agonist 2-Chloro-N6-cyclopentyladenosine (CCPA), while nodose C-fiber could be activated by both A1 receptor agonist CCPA and A2A receptor agonist CGS-21680. Similar to most mediators discussed above, adenosine did not activate esophageal nodose Aδ-fibers [25]. Moreover, adenosine A2A receptor agonist CGS-2168 also significantly increased esophageal distension-evoked action potential discharges in esophageal nodose C-fibers. Such mechanical sensitization effect could be abolished by selective A2A receptor antagonist SCH58261, and by TRPA1 antagonists HC-030031 and AP18 [26].

### 5.7. S1P

Sphingosine-1-phosphate (S1P) is a metabolite of sphingolipid that is released by different types of cells in the extracellular space at the site of inflammation. S1P has been reported to participate in allergic inflammation-induced airway hypersensitivity through the S1P3 receptor that is expressed in vagal nodose neurons [58,59]. The effects of S1P on esophageal nodose and jugular C-fibers have been investigated. S1P was able to evoke action potential discharges in jugular C-fibers at their nerve terminals in the esophagus but failed to activate esophageal nodose C-fibers. S1P receptors 1, 2, and 3 were identified in esophageal-labeled nodose and jugular neurons. S1P receptor 1 and 3 agonists each partially mimicked the S1P-elicited effect in jugular C-fibers. S1P did not appear to non-selectively sensitize esophageal nodose or jugular C-fibers to other activating stimuli, such as mechanical distension [28]. It is of considerable interest to further clarify whether S1P may contribute to esophageal dysfunctions in certain esophageal inflammatory conditions, such as eosinophilic esophagitis.

## 6. Conclusions

TRPV1 plays an important role in processing noxious stimuli in nociceptive afferent neurons [60]. TRPV1 can be activated not only by capsaicin but also by acid (and heat), making it a viable target to inhibit acid reflux-induced esophageal nociception. TRPV1 is richly expressed in polymodal afferent C-fibers in the esophagus. There are at least three major subtypes of these afferent nerves, namely vagal nodose C-fibers, vagal jugular C-fibers, and spinal DRG C-fibers. All three subtypes have the characteristics of classical nociceptors. In healthy conditions, they serve to help “sense” the internal environment of the esophagus, where they are somewhat selectively activated by noxious and potentially dangerous stimuli. The consequential action potential discharge signals to the CNS then provide warning sensations, such as pain and heartburn, as well as subconscious autonomic reflexes, such as an increase in secretions. In this manner, these afferent nerves play a role in host defense.

In the inflamed esophagus, tissue-released mediators can induce excessive stimulation of C-fibers. This may lead to peripheral and central sensitization and produce chronic warning sensations of pain/heartburn and reflexes that extend far beyond any useful function. Despite the significant progress that has been made in the management of gastroesophageal reflux symptoms, there are still 30–40% of patients with persistent symptoms that are refractory to proton pump inhibitors [61,62]. A better understanding of the function of each nociceptor subtype, along with the mechanisms underlying their activation and sensitization, may lead to novel therapeutic strategies aimed at reducing the suffering that accompanies acid reflux disorders. Such understanding may also lead to strategies that more readily allow for the distinction between cardiac and non-cardiac chest pain.
